# Harnessing the role of mitogen-activated protein kinases against abiotic stresses in plants

**DOI:** 10.3389/fpls.2023.932923

**Published:** 2023-02-24

**Authors:** Yasir Majeed, Xi Zhu, Ning Zhang, Noor ul-Ain, Ali Raza, Fasih Ullah Haider, Huaijun Si

**Affiliations:** ^1^ College of Agronomy, Gansu Agricultural University, Lanzhou, China; ^2^ State Key Laboratory of Aridland Crop Science, Gansu Agricultural University, Lanzhou, China; ^3^ College of Life Science and Technology, Gansu Agricultural University, Lanzhou, China; ^4^ Fujian Agricultural and Forestry University (FAFU) and University of Illinois Urbana-Champaign-School of Integrative Biology (UIUC-SIB) Joint Center for Genomics and Biotechnology, Fujian Agriculture and Forestry University, Fuzhou, China; ^5^ College of Agriculture, Fujian Agriculture and Forestry University (FAFU), Fuzhou, Fujian, China; ^6^ College of Resources and Environmental Sciences, Gansu Agricultural University, Lanzhou, China

**Keywords:** abiotic stresses, climate change, plant physiology, signaling pathway, stress tolerance, transcription factor

## Abstract

Crop plants are vulnerable to various biotic and abiotic stresses, whereas plants tend to retain their physiological mechanisms by evolving cellular regulation. To mitigate the adverse effects of abiotic stresses, many defense mechanisms are induced in plants. One of these mechanisms is the mitogen-activated protein kinase (MAPK) cascade, a signaling pathway used in the transduction of extracellular stimuli into intercellular responses. This stress signaling pathway is activated by a series of responses involving MAPKKKs→MAPKKs→MAPKs, consisting of interacting proteins, and their functions depend on the collaboration and activation of one another by phosphorylation. These proteins are key regulators of MAPK in various crop plants under abiotic stress conditions and also related to hormonal responses. It is revealed that in response to stress signaling, MAPKs are characterized as multigenic families and elaborate the specific stimuli transformation as well as the antioxidant regulation system. This pathway is directed by the framework of proteins and stopping domains confer the related associates with unique structure and functions. Early studies of plant MAPKs focused on their functions in model plants. Based on the results of whole-genome sequencing, many MAPKs have been identified in plants, such as *Arbodiposis*, tomato, potato, alfalfa, poplar, rice, wheat, maize, and apple. In this review, we summarized the recent work on MAPK response to abiotic stress and the classification of MAPK cascade in crop plants. Moreover, we highlighted the modern research methodologies such as transcriptomics, proteomics, CRISPR/Cas technology, and epigenetic studies, which proposed, identified, and characterized the novel genes associated with MAPKs and their role in plants under abiotic stress conditions. *In-silico*-based identification of novel *MAPK* genes also facilitates future research on MAPK cascade identification and function in crop plants under various stress conditions.

## 1 Introduction

One of the sustainable development goals is to end world hunger, and feeding a growing population is a significant worldwide societal concern (Raza et al., 2021; Farooq et al., 2022; Rivero et al., 2022). Despite the world’s population doubling, the long-term drop in global undernourishment has been caused by a substantial rise in food availability since 1960 (Ritchie and Roser, 2020). Nevertheless, there are currently more than 820 million hungry people around the globe (FAO et al., 2019). Only 9% of the world’s agricultural land is suitable for growing crops, while the remaining 91% is subjected to abiotic stress, which frequently occurs in combination. Abiotic stresses cause losses in agricultural productivity of more than 50% (Raza, 2021; Rivero et al., 2022). Still, due to climate change and the overuse of natural resources, their severity and adverse effects are anticipated to increase significantly, which not only reduce crop production but also cause food insecurity in the near future (Minhas et al., 2017; Farooq et al., 2022; Rivero et al., 2022).

Due to industrialization and climate change in recent decades, plants are normally exposed to various abiotic stresses such as drought, salinity, extreme temperature ranges, nutrient deficiency, high heavy metal concentrations, and osmosis stress (Haider et al., 2021; Raza et al., 2021; Raza et al., 2022a; Raza et al., 2022b; Raza et al., 2022c; Raza et al., 2022d; Raza et al., 2022e). They cause a lot of damage to plants’ physiology and also reduce growth and development that ultimately minimizes the productivity of crop plants (Anjum et al., 2017). To ensure good crop growth and optimum productivity, stress tolerance mechanisms are imperative to be studied for combating abiotic stresses in crop plants (Baer-Nawrocka and Sadowski, 2019; Raza et al., 2022a; Raza et al., 2022b; Raza et al., 2022c). Understanding these stress tolerance mechanisms will enable the generation of more climate-smart and stress-tolerant lines, which will maintain stability in the growth and productivity of agricultural productivity. For this, it is necessary to understand the genetic basis of a plant’s interaction when encountering ecological stress. Several studies considering transcriptomics, genomics, proteomics, metabolomics, and genome editing *via* CRISPR/Cas technology provide a roadmap toward the acclimatization mechanism in plants and crops (Wang et al., 2019; Raza et al., 2022a; Raza et al., 2022b; Raza et al., 2022c; Raza et al., 2022d; Yaqoob et al., 2023). In-depth molecular studies aid us in developing varieties and cultivars through biotechnology, genetic engineering, and other advanced breeding methods to develop plants that could adapt to different abiotic stresses in a short time (Popescu et al., 2009; Raza et al., 2022a; Raza et al., 2022b; Yaqoob et al., 2023). Post-translational modification and signal transduction are mediated by a process called phosphorylation, which changes the expression of genes through transmission of protein signals. A serine/threonine-protein kinase family called mitogen-activated protein kinase (MAPK) is one of the widely studied gene families and contributes to plant productivity under fluctuating environmental conditions (Hamel et al., 2006).

In this regard, one of the major signal transduction pathways that transduce extracellular stimuli into intracellular responses related to stress is mitogen-activated protein kinase (Mathobo et al., 2017). The first MAPK encoding gene was cloned in the 1990s; to date, many MAPK genes have been identified and isolated from different plants (Zaıdi et al., 2010), which are activated under abiotic stresses like *AtMPK4* and *AtMPK6* in *Arabidopsis* and in rice (*Oryza sativa*) and *OsMAPK5* and *OsMAPK2* under drought stress (Nadarajah and Sidek, 2010). The abscisic acid (ABA) signaling pathway regulates plant growth and development under abiotic stress conditions, such as drought or high salinity (Cutler et al., 2010). Phosphorylation of two ABA-responsive transcription factors (*ABF1* and *ABF4*) by *AtCPK4*, *11*, and *32* suggested the role of kinases in regulating ABA signaling through these transcription factors under stress conditions (Choi et al., 2005). Using a yeast two-hybrid test, researchers looked at the interactions between 30 members of the MPK family, 9 *CPKs*, 8 *PP2Cs*, 5 *SnRKs*, and 8 *PP2Cs* in maize’s (*Zea mays*) MAPK signaling pathways. Moreover, three *ZmCPKs* connect with three distinct *ZmSnRK* members, whereas four *ZmCPK* members positively interact with 13 different *ZmMPK* members in various combinations. These four *ZmCPK* proteins originate from three distinct maize groupings. These physical connections between *ZmCPKs*, *ZmSnRKs*, and *ZmMPKs* revealed that these signaling pathways might interact directly with the defense mechanism in maize and have indirect effects. The current work might contribute to a better understanding of plant signal transduction (Khalid et al., 2019). Concerning the downregulation of *ZmMPK5*, due to *ZmCPK11* silencing, the role of *ZmCPK11* upstream of *ZmMPK5* has been proposed (Ding et al., 2013).

**Figure 1:**
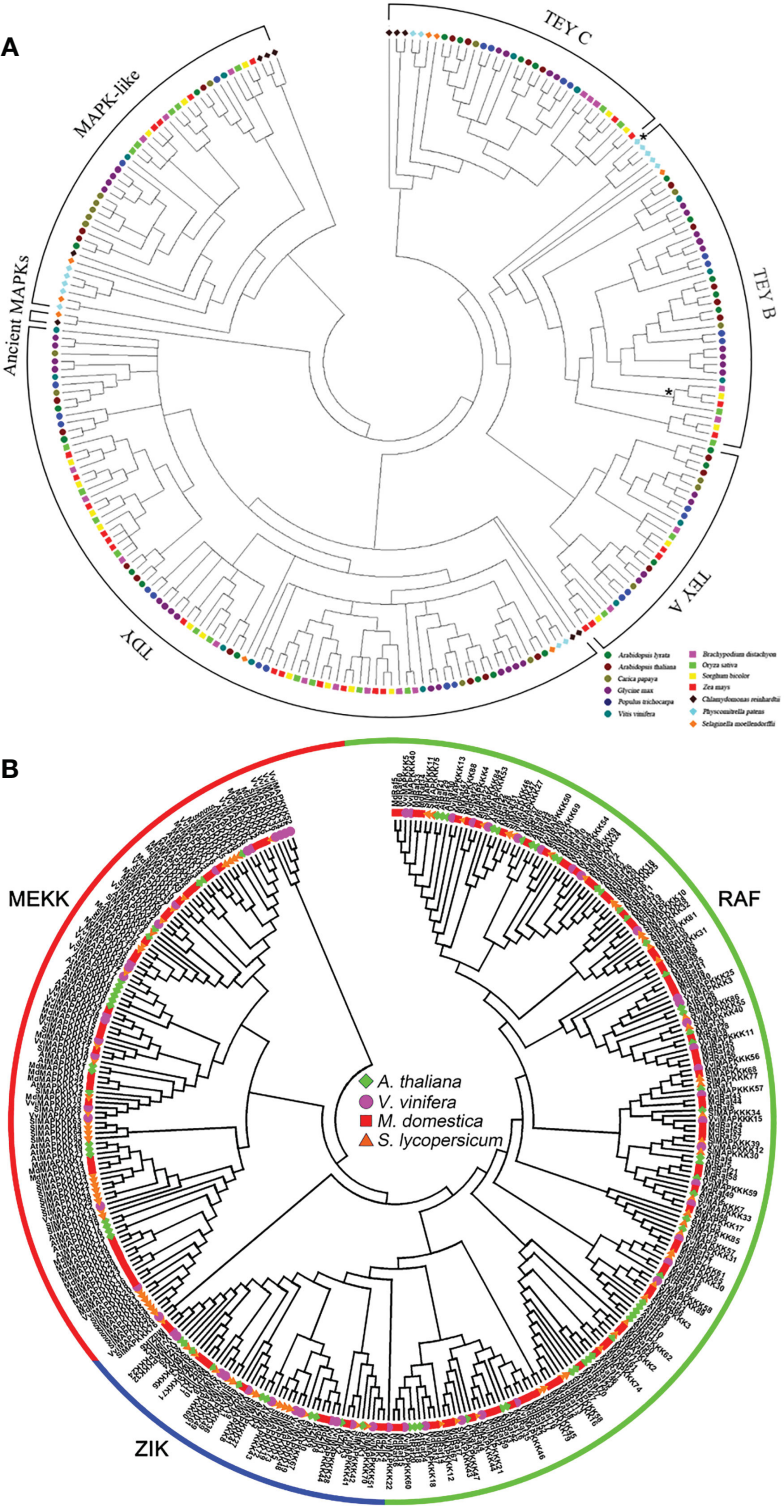
**(A)** Phylogenetic analysis of plant mitogen-activated protein kinases (MAPKs) and MAPK-likes in 13 plant species following Bayesian inference. Different colors indicate different species. The monocot clade and the PP sequence with the atypical MEY activation loop within the TEY-B clade are marked by asterisks. **(B)** Phylogenetic analysis of MAPKKKs in grapevine, apple, tomato, and *Arabidopsis* using the neighbor-joining (NJ) tree using MEGA-X with 1,000 bootstraps. Adapted from He et al. (2020) and Janitza et al. (2012), open-access articles distributed under the terms of the Creative Commons Attribution License (CC BY).

MAPK gene families consist of a vast number of genes that are classified into four different groups: A, B, C, and D. Owing to the evolutionary divergence in different plants, these groups contain a different number of MAPK genes; some of them are listed in **Figure 1** and **Table 1**. This MAPK signaling cascade works like a chain reaction as mitogen-activated protein kinsase kinase (MAPKK) is activated by the upstream of the mitogen-activated kinase (MAPKKK), which, in turn, activates the mitogen-activated protein kinase (MAPK) (Wang et al., 2014; Mitula et al., 2015). This full chain of MAPKs is conserved in plants, which signifies the evolutionary perspective of MAPKs. One of the most important methods that is triggered by posttranslational modification of signal transduction is called phosphorylation (Wang et al., 2019). MAPKKK phosphorylates MAPKK on the conserved serine/threonine motifs (Rodriguez et al., 2010), which finally brings about the phosphorylation of TXY (T is threonine, Y is tyrosine, and X is any amino acid) in MAPKs (Taj et al., 2010).

**Table 1:** Identification of groups of MAPK gene families in different plant species.

Plant species	Group A	Group B	Group C	Group D	Total	References
*Arabidopsis*	3	5	4	8	20	Cçakır and Kılıcçkaya (2015)
Rice	2	1	2	10	15	Reyna and Yang (2006)
Wheat	11	13	22	63	109	Lian et al. (2012)
Maize	7	7	6	19	39	Wang et al. (2010)
Cotton	4	6	5	12	27	Wang et al. (2016)
Poplar	4	4	4	9	21	Zhao et al. (2018)
Tomato	3	4	2	7	16	Kong et al. (2012)
Apple	5	6	5	10	26	Zhang et al. (2013)
Potato	1	4	2	8	15	Zaynab et al. (2021)

Transcription factors and downstream kinases activate the cell after receiving extracellular signals from the activated MAPKs, which describe the variation of the cellular development factors (Zhang et al., 2018). The process of transmission and amplification of signals occurs in stepwise phosphorylation (Hamel et al., 2006). When plants are encountered by any abiotic stress or wounding, MAPK, as well as other hormones like ethylene, jasmonic acid, and salicylic acid, are activated. Studies also suggested that pathogen stimuli also cause the induction of MAPKs in various plants like alfalfa (*Medicago sativa*), rice (*Orzya sativa*), maize (*Z. mays*), and potato (*Solanum tuberosum*) (Andrasi et al., 2019). Under adverse environmental conditions like high temperature and water scarcity, MAPKs play a key role in signal transduction (Muhammad et al., 2019). Studies also have revealed that MAPK cascade is also activated under salt stress and freezing temperature (Teige et al., 2004). Shreds of evidence provide the signaling activation of MAPKs during early wounding in different plants such as *Arbodiposis*, apple (*Malus hupehensis*), poplar (*Populous alba*), and rice, as reported previously (Takahashi et al., 2011). Recently, many *MAPK* genes have been identified, which play a role under various stress conditions, but there is little knowledge on the molecular mechanisms of MAPK triggering and signaling (**Figure 1**) (Mohanta et al., 2015). The available knowledge about the molecular characterization of the *MAPK* gene family under abiotic stress is summarized owing to need for more information about MAPK signaling and triggering in many crops. In this review, we will focus on the action of transcription factors, transcriptomic studies, and the molecular basis for understanding the biological, biochemical, and physiological processes of different plants under abiotic stresses. Furthermore, we summarized the classification of different MAPK gene families in response to abiotic stress in plants and molecular and cellular signaling pathways in plants for acclimatization under an adverse environment. After all, many phosphorylated MAPKs play a role in signal transmission. In short, incorporating MAPK gene families in plant breeding to produce stress-smart varieties is another aspect of research in MAPKs. This review may help to comprehend the ecological importance of MAPKs in plants to overcome the abiotic stresses for sustainable crop production and also provide new insights for breeders to incorporate *MAPK* gene families in plant breeding program to produce abiotic stress resistance varieties.

## 2 Mechanism of MAPKs in crop plants

Plants have acquired different acclimatization strategies under harsh environmental conditions over time *via* a number of molecular systems that consist of sensing, signaling, and expression under stress conditions by stress-responsive genes (Mahmood et al., 2020). MAPKs are one of the tools that regulate growth and development, cell division, proliferation, apoptosis, hormonal response, and other stress responses by an extremely conserved network. It consists of the three protein kinases MAPK, MAPKK, and MAPKKK. Sequential phosphorylation activates these cascades such as activation of MAPKKK phosphorylates the S/T-X (S/T is a serine/threonine and X is an arbitrary amino acid) conserved motif that is present in the activation loop of MAPKK (Rodriguez et al., 2010); then, the activation of MAPKK phosphorylates the T-X-Y (T is threonine, Y is tyrosine, and X is any amino acid) in the variant motif present in the activation loop of MAPK (Taj et al., 2010). Then, these MAPK cascades send the message in a well-designed manner to the primary genes responsive to tolerance and then to secondary genes, which induce tolerance in crops under stress conditions (Hamel et al., 2006). The MAPK cascades are also phosphorylated by the following activities such as regulation of microtubule proteins, cytoskeletal activities, and other transcription factors that help in numerous responses and phospholipases (Danquah et al., 2014). The transmission of extracellular stimuli into cells and downstream kinases is also activated by MAPK cascades (Xu et al., 2010). The main channel of signal transduction and post-transcriptional modification is carried out by phosphorylation. The process of phosphorylation is a post-translational modification process that alters the expression of downstream genes as well as diffuses and intensifies the external signals (Colcombet and Hirt, 2008).

**Figure 2:**
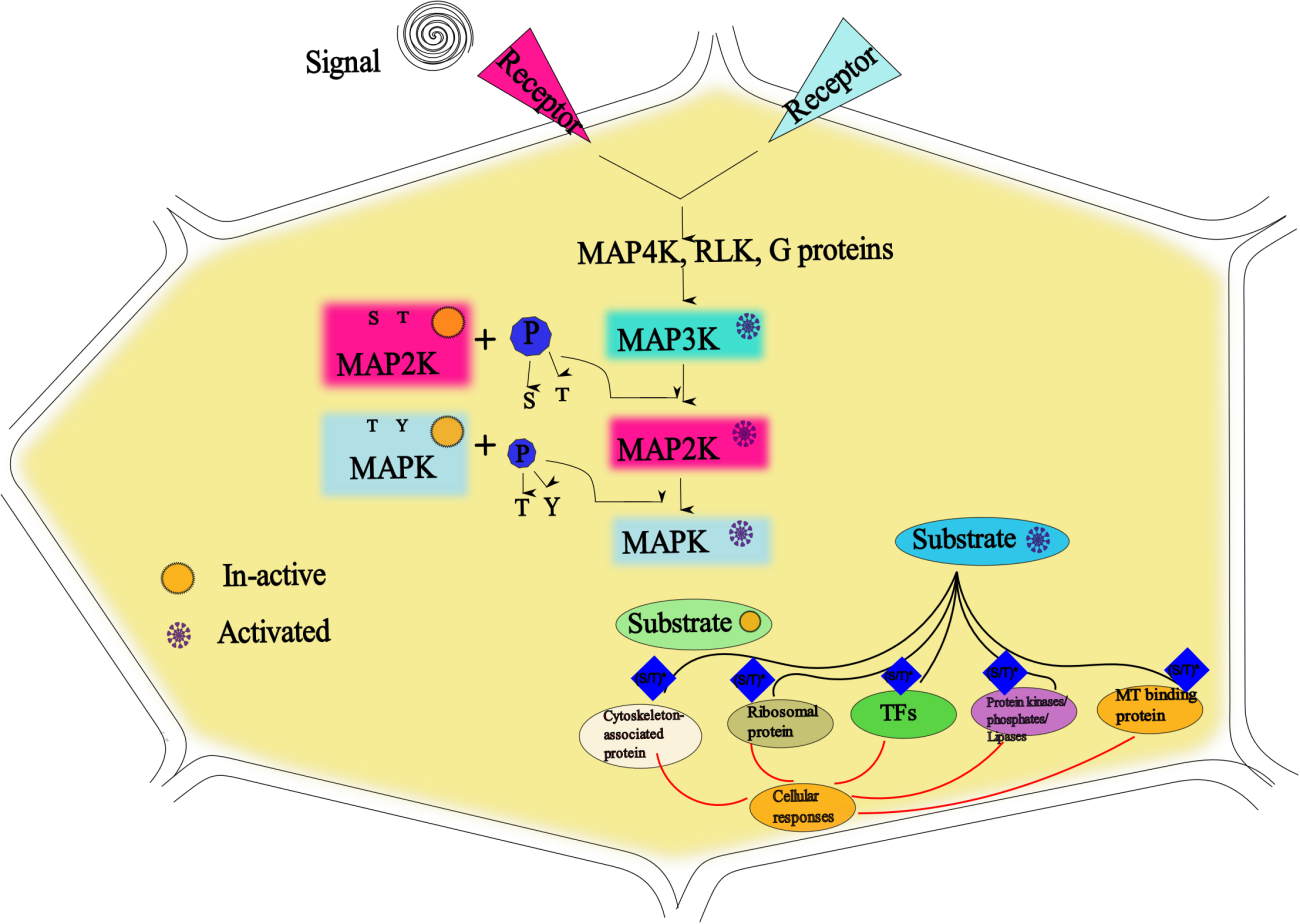
The receptors after receiving the signals activate the specific mitogen-activated protein kinase (MAPK) proteins that are inactive before. Subsequently, MAP4K activates other MAPKs (including MAP2K and MAP3K) by phosphorylating the ST (S/T is serine/threonine) and TXY (T is threonine, Y is tyrosine, and X is any amino acid) motif in MAPKs (Rodriguez et al., 2010; Taj et al., 2010). As a result, MAPKs activate the transcription factors (TFs), enzymes, and other downstream kinases that transmit extracellular environmental signals to the cells that play a role in growth, development, stress response, and other physiological and biochemical processes (Zhang et al., 2018).

One of the wide-ranging gene families is the serine/threonine protein kinase family of MAPKs, which are protein kinases and are enzymatic in nature and mediate phosphorylation (Rodriguez et al., 2010; Xu et al., 2010). Conventionally, MAPK cascades transmit signals downstream by the activation of the stimulated receptors of the cell membrane (Wang et al., 2014). By following this mechanism, MAPKs, after activation, control the expression of many genes and proteins by the phosphorylation of transcription factors. This mechanism in plants plays a key role in cell differentiation, cell growth, development, and hormonal movement, and in response to various biotic and abiotic stresses (Komis et al., 2018). In crop species, the MAPK gene families are recognized owing to the many tolerant genes in abiotic stress response, evident from genetic studies of MAPK activity in recent years (SŠamajovaí et al., 2013). The MAPK mechanism is depicted in **Figure 2**, where extracellular signals are detected by a plasma membrane and by sensors in cytoplasm, which act like a chain reaction, leading to the activation of MAPKKK and, subsequently, MAPKK and, as a result, phosphorylation of MAPK, which helps in the phosphorylation of proteins, enzymes, and post-transcriptional factors in the nucleus. These factors finally send a message to stress-tolerant responsive genes (Bigeard and Hirt, 2018; Singh et al., 2019). By understanding this novel mechanism, MAPK genes can be identified in different crop plants, which may help in maintaining plant growth under various abiotic stress conditions, but still, there is a limitation due to the activation of other intricate stress responsive mechanisms.

## 3 MAPK complexity in abiotic stress signaling interaction

Abiotic stresses, such as drought, cold, heat, salinity, and metals, are closely associated with an adverse influence on the physiology of plants (Krasensky and Jonak, 2012; Raza, 2021; Raza et al., 2021; Raza et al., 2022a; Raza et al., 2022b; Raza et al., 2022c; Raza et al., 2022d). For example, when the temperature rises, water deficiency, salinity, and osmotic pressure in the tissues of plants may be encountered (Bita and Gerats, 2013). Likewise, due to salinity and water scarcity, plants suffer from osmotic stress, and signaling molecules are activated in response to stress conditions (Rengasamy, 2006). The mechanism of expression of resistance genes in various plants like maize (*Z. mays*), rice (*O. sativa*), potato (*S. tuberosum*), and *Arabidopsis* is regulated under various abiotic stress conditions, which are activated by messenger-mediated signal transduction (Zhu, 2016; Zandalinas et al., 2019). The large numbers of stress-responsive genes like MAPK genes or MAPK cascades participate in a variety of abiotic stresses for the protection and survival of plants (Ji et al., 2017; Yanagawa et al., 2017). MAPK gene families have multiple functions in plants, such as growth and development, immune defense system, and response to biotic and abiotic stresses. Certain studies also point toward the role of MAPK cascade in the regulation of cell death and defense responses (Qiu et al., 2008; Zhou et al., 2017).

Recently, it was also reported that MAPK cascade has a dual function in plant immunity: the basal resistance is regulated positively and immunity regulation is facilitated negatively by kinase protein (Zhang et al., 2012). One case reported in *Arabidopsis* also provides evidence that MAPKs play a vital role in pathogen signaling (Doczi et al., 2007). Furthermore, MAPK cascade mediates Ca^2+^ reactive oxygen species (ROS) by signaling during early wounding (Pitzschke and Hirt, 2009). MAPK pathways regulate the synthesis of ROS, and certain genes of MAPKs are induced by ROS generation (Colcombet and Hirt, 2008). H_2_O_2_ also plays a key role in activating orthologs of MAPKs in many crops like tobacco (*Nicotiana tobacum*) and *Arabidopsis* (Xing et al., 2008). MAPK cascade in *Arabidopsis* regulates H_2_O_2_ metabolism (Nakagami et al., 2006), and in tobacco, it is an important component in ROS metabolism (Ning et al., 2010). In maize, H_2_O_2_ induces transcription and expression of MAPK cascade, whereas in tobacco, it protects against ROS-mediated injury under osmotic stress (Zhang et al., 2010; Kong et al., 2011). This is due to the different expression patterns of MAPK gene families under different stresses in different plant species. In maize, the MAPK network works as an early signaling response by regulating the production of ROS in plants subjected to drought stress (Gao and Xiang, 2008).

In later developmental stages, one MAPKKK gene called *MAPKKK20* in *Arabidopsis* improves salt tolerance (Jammes et al., 2009). Similarly, water loss by transpiration in the *Arabidopsis* double mutant named *MPK9/MPK12* is less as compared to its wild type. Moreover, it was also documented that ABA regulates the physiological response under abiotic stress (Tajdel et al., 2016). In *Arabidopsis*, one MAPKKK gene named *MAPKKK18* shows reduced stomatal opening under normal conditions in mutant plants (Mitula et al., 2015). Furthermore, this mutant also shows ABA-induced stomatal closure, which shows that *MAPKKK18* is directly interacting with ABA components, which plays a key role in signal modulation as SnRK2-6 kinase and PP2C phosphatase ABI1 (Zhang et al., 2006). An increment of ABA in plants is an indication of oxidative stress. An abnormal level of ROS causes the oxidation of free radicals such as hydrogen peroxides, which is called oxidative stress and leads to the injury of cells and tissues. The *MAPK* gene families reinfoirces the ABA-induced antioxidant defense system by decreasing the ROS production in many crops like maize (Shi et al., 2011).

Under physiological and biochemical conditions of abiotic stresses, the intricate role of kinase proteins by signal transduction of MAPKs still has research gaps. It is required to associate the link between MAPKs and their corresponding stress *in vivo.* In the future, further functional analysis on MAPK members for physiological and biochemical roles in stress management can be helpful in breeding programs for innovation and advancement of agricultural science.

## 4 Role of MAPKs under abiotic stresses

Plants often experience various abiotic stresses (drought, low and high temperature, salinity, osmotic, etc.), which significantly affect their growth, productivity, and nutritional quality (Yu et al., 2019; Sun et al., 2020; Raza, 2021; Raza et al., 2021; Raza et al., 2022a; Raza et al., 2022b; Raza et al., 2022c; Raza et al., 2022d). The role of MAPKs against various abiotic stresses are briefly discussed in the subsequent sections.

### 4.1 The role of MAPKs under drought and oxidative stress

Drought stress is a major environmental factor affecting crops’ growth and productivity, leading to significant socioeconomic damage (Sinha et al., 2011; Zhang et al., 2018; Raza et al., 2022a). Drought occurs when water uptake within the plant by root is reduced due to low moisture in the soil, afflicting root morphology growth and physiology owing to wilt conditions, and consequently reducing the annual yield in plants (Aditi et al., 2020; Ahmad et al., 2021; Feng et al., 2022; Lu et al., 2022; Raza et al., 2022a). Biochemical and transcriptional studies have elaborated the MAPK response in drought in grassy and woody plants (Huang et al., 2020). Recently, it was studied that the MAPK cascade was induced with *Arbuscular mycorrhizal* fungi (AMF) inoculation in apple (*Malus hupehensis*) seedling following increased expression level of MAPKs such as *MdMAPK16-2*, *MdMAPK17*, and *MdMAPK20-1* by 36.93%, 58.14%, and 54.14%, respectively, compared to those that do not have AMF inoculation (Xu and Chua, 2012). By regulating the RNA de-capping process in *Arabidopsis*, *MAK6* improves the tolerance to dehydration (Hua et al., 2006). In *Arabidopsis*, MAPK gene families are activated. The promoter *RD29*, which is a dehydration-responsive gene *via* transient expression assay, suggests that MAPK cascade plays a vital role in drought signaling (Zaheer and Akhtar, 2016). In *Arabidopsis*, the transcriptional regulation of 44 MAPKs has been identified, out of which some are induced by water stress such as *MPK2*, *MPK4*, *MPK5*, *MPK12*, and *MAPKKK4* (Moustafa et al., 2014).

**Figure 3:**
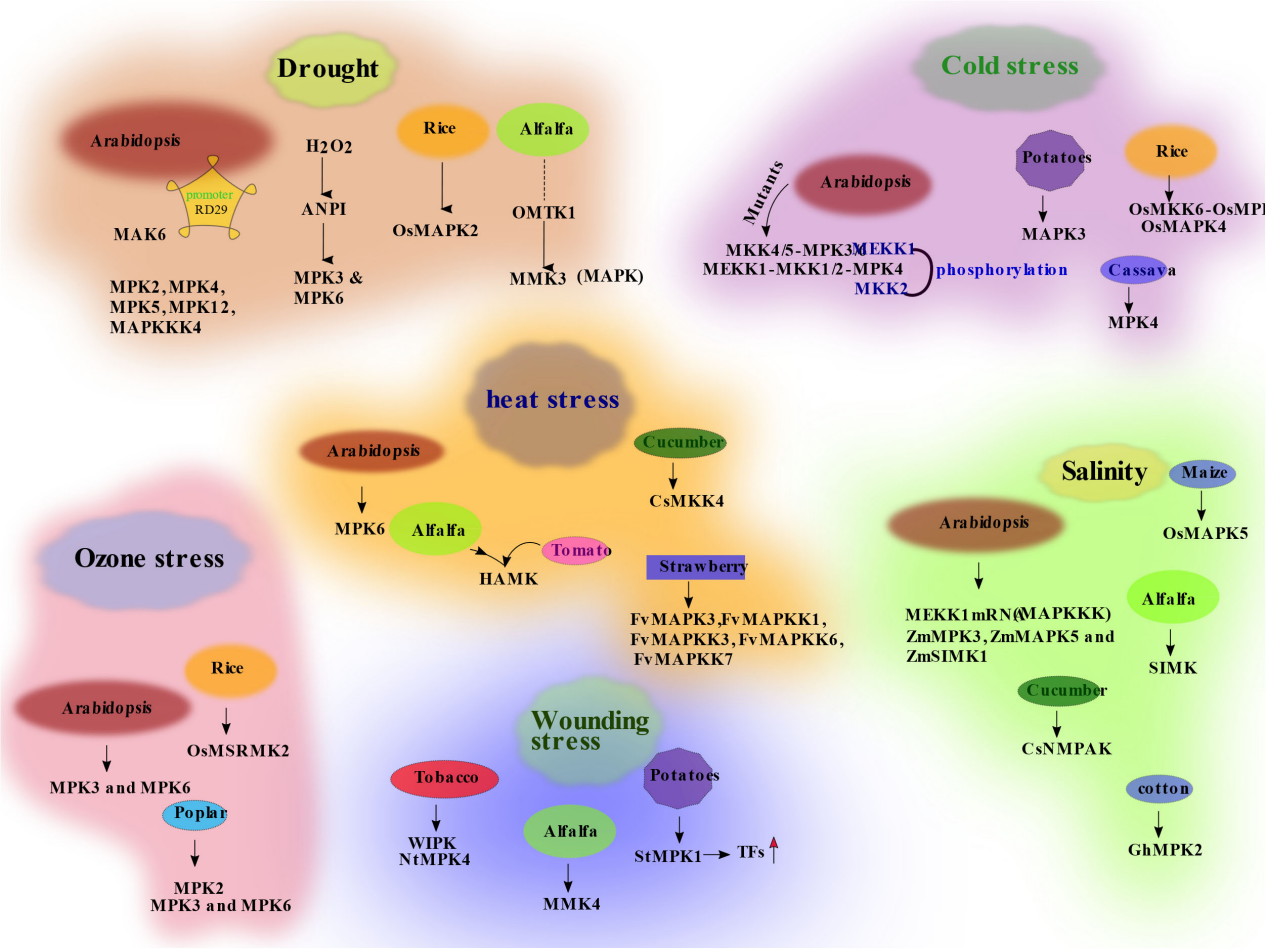
The schematic diagram of different mitogen-activated protein kinase (MAPK) signaling molecules under different stress conditions in different crops. Solid arrows show verified pathways; dashed arrows indicate assumed pathways; question marks indicate unknown cascade components.

Potato (*S. tuberosum* L.) is known as one of the drought-sensitive crops, and serious yield loss has been threatened by drought stress (Handayani et al., 2019; Sattar et al., 2021), as well as lessened the quality of potato crops (Iftikhar et al., 2017). Studies revealed that 108 MAPK protein-coding genes have been found in potatoes (Zhu et al., 2021). More recent studies confirmed that 22 MAPK genes had been reported in the potato genome, such as *StMAPK1* to *StMAPK22* (Zaynab et al., 2021; Zhu et al., 2021), of which, six MAPKs have been related to abiotic stress, as well as six types of plants hormones (Boguszewska-Mankowska et al., 2020). *StMAPK11* response is well studied for drought stress in potato, and it shows reasonable drought tolerance in potato when subjected under drought conditions (Zhu et al., 2021). The drought sensitivity of potato is due to the shallow root system that cannot explore the moisture from the deeper soil layers (Kumar et al., 2008). Further studies related to the characterization of MAPKs in drought stress can help improve the gene expression and physiology of potato. Many MAPKs related to drought stress tolerance are also observed in rice such as MAPKKK protein drought-hypersensitive mutant1 (DSM1), which shows reduced ROS generation under water-stressed conditions and increases the survival of plants under drought stress (Gao and Xiang, 2008). Overexpression of *OsMAPK5*, which is an ortholog of *Arabidopsis* MAPK3, also acts as a drought-responsive gene. Studies also suggested that the *OsMAPK2* responds to drought stress and salt stress signaling within 15 min in rice crops, as shown in **Figure 3** (Popescu et al., 2009).

Mostly abiotic stresses such as drought, cold, heat, and osmotic stress interrupt the metabolic equilibrium of the cell, which causes oxidative stress (Gill and Tuteja, 2010; Mittler et al., 2022; Raza et al., 2022b). The abnormal level of ROS such as free radicals and non-radicals leads to damage of specific molecules, which injured the cells or tissues in broad terms. It is called oxidative stress, which is due to the oxidation of molecules (Xing et al., 2007; Mittler et al., 2022). Antioxidants like endogenous or exogenous compounds help in the removal of ROS. Scavenger enzymes in plants like catalase decompose H_2_O_2_ and overcome oxidative stress. ABA regulates the *A. thaliana* CAT1, and *MAPKK* inhibitor PD98059 delayed CAT1 expression, which is mediated by ABA signaling (Ning et al., 2010). Under dehydration stress, the *MKK1* and *MPK6* mutant in *A. thaliana* alter their response to ABA. It was proposed from the above findings that *MKKI–MPK6* controls the metabolism of H_2_O_2_ by CAT1 with the absence of ABA-mediated activation of *MPK6* and *MMKI* (Nakagami et al., 2006). Plant defense mechanism and salicylic acid (SA) accumulation are controlled by the CAT2 expression, which is stimulated by *MEKK1* and *MPK4* (Colcombet and Hirt, 2008). ROS metabolism is regulated by the *MEKK1–MPK4* pathways (Ning et al., 2010). Many other MPAKKKs in *A. thaliana* are activated by H_2_O_2_, such as ANPI, which causes the downstream activation of *MPK3* and *MPK6*, as shown in **Figure 3** (Ding et al., 2009). These findings suggest that MAPKs not only are induced by ROS but also control ROS and arbitrate oxidative stress in crops. Oxidative stress is a communal response under abiotic and biotic stress as ROS is a conjunction point to indicate the stress in plants. Recently, ROS-mediated MAPK signaling in plants has been described. In *A. thaliana* mutants, MAPK studies reveal the specific protein association related to ROS control (Ning et al., 2010). The significant roles of *MAPK* genes in different crops like alfalfa (*M. sativa*), rice (*O. sativa*), cotton (*Gossypium hirsutum*), maize (*Z. mays*), apple (*Malus hupehensis*), potato (*S. tuberosum*), cucumber (*Cucumis sativus*), strawberry (*Fragaria vesca*), mulberry (*Moraceaemorus*), and cassava (*Manihot esculenta*) under drought condition are mentioned in **Table 2**. From the above discussion, it is concluded that many *MAPK* gene families in different crops integrate stress-related proteins and regulate stimulus response. It is necessary to identify and isolate more MAPK genes for crop improvement.

**Table 2:** MAPK gene signaling responses in different plants under drought stress conditions.

Plant species	*MAPK* genes	Activation	Plant stage	References
*Arabidopsis*	*AtMPK2, AtMPK3, AtMPK4, AtMPK5, AtMPK12*, and *AtMKK*	Activated at the transcriptional level Activated by promoter RD29 under dehydration	Seedling	Chen et al. (2015)
Alfalfa	*MsP44, MsMKK4*	Activated at the post-transcriptional level	Seedlings	Chen et al. (2015)
Rice	*OsMPK3,4*	Activated by the regulation of protein DSM1 (drought-sensitive mutant1)	Seedling	Huang et al. (2020)
	*OsMKK6*	Transcriptionally regulated by drought	Seedling	Huang et al. (2020)
	*OsDSM1*			Gao and Xiang (2008)
	*OsMSRMK2,5* and *OsMAPKK1*	Activated under combined drought and osmotic stress	Seedling	Wang et al. (2010); Chen et al. (2015)
Cotton	*GhMAPK2, GhMAPK16*	Activated under combined drought and osmotic stress	Germination	Wang et al. (2007)
Maize	*ZmMPK2*	Activated under reducing water and osmotic pressure	Seedling	Zhang et al. (2011)
Apple	*MaMPK*	Activated at the transcriptional level	Seedling	Sun et al. (2017)
Potato	*StMAPK11*	Activated by induction of drought and hormone stress	Flowering	Zhu et al. (2021)
Cucumber	*CsMAPKs*	Activated by the regulation of stress-associated genes and drought stress	Flowering	Wei et al. (2014)
Strawberry	*FvMAPK5,8*	Activated at the transcriptional level	Reproductive	Zhou et al. (2014)
Mulberry	*MnMAPK1, MnMAPK2*	Negatively activated by drought stress	Reproductive	Yan et al. (2016)
Cassava	*MeMAPK*	Activated 20% in leaves and 70% in roots under high drought conditions	Flowering	Chinnusamy et al. (2007)

### 4.2 The role of MAPKs under cold stress

One of the most critical factors that affect the growth and development of crops is cold stress or chilling temperatures like sudden frost and snow, and freezing temperatures cause serious damage to crop production and quality (Raza et al., 2021; Ma et al., 2022; Raza et al., 2022c). When plants survive under cold or freezing temperatures, it is called cold tolerance or winter hardiness (Peng et al., 2006). The physiological and metabolic status of crops is changed by the altered expression of many genes during cold acclimatization (Agarwal et al., 2010; Medina et al., 2016; Raza et al., 2021; Raza et al., 2022a; Raza et al., 2022c). In *Arabidopsis*, MAPK genes like *MEKK1, MKK2*, *MPK4*, and *MPK6* were shown to be activated under cold stress (Teige et al., 2004). The cold-sensitive gene *MKK2* does not have any effect on *MPK4* or *MPK6*, which suggests that *MKK2* is an upregulator of *MPK4* and *MPK6* in low temperature. This outcome shows that chilling temperature activates *MEKK1*, *MPK3*, *MPK4*, and *MPK6* (Krasensky and Jonak, 2012). Another MAPKK gene named *SbMAPKK* in halophytes, such as sea bean (*Salicornia brachiate*), shows upregulation under cold stress (Meng and Zhang, 2013), because cold as well as salt stress induces some kinase molecules that allow plants to survive under adverse environments.

Recently, in tomato, CRISPR/Cas9-mediated *SlMAPK3* mutants were used to investigate the relationship between ferulic acid (FA) and *SlMAPK3* under chilling temperatures. It was shown that under low temperature, i.e., 4°C, FA content increases, which then increases the FA synthesis-related gene (*SlPAL5*, *SlC3H*, and *SlCOMT*) expression. However, the knockout of *SlMAPK3* inhibited the content of FA and the expression of those genes compared with the control, which suggested a close relationship between *SlMAPK3* and FA. Plant response to cold stress depends heavily on the CBF/DREB1 (C-repeat binding factor/dehydration resistance element binding protein 1)- and ICE1 (inducer of CBF expression1)-dependent transcriptional regulatory mechanisms. It has been demonstrated that CBFs may bind to cis elements in the COR (COLD RESPONSIVE) gene promoters, sufficiently activating the expression of *COR* genes and inducing resistance to cold stress. It was revealed that the FA in tomato fruit provides resistance to chilling stress by upregulating the gene expression of the repeat binding transcription factor (CBF), a transcriptional pathway, in a MAPK3-dependent manner (Shu et al., 2022). Under cold stress, *MKK4/5-MPK3/6* and *MEKK1-MKK1/2-MPK4*, the two MAPK gene mutants, show response to external stimuli produced by cold stress (Furuya et al., 2013; Pitzschke et al., 2014). It is also documented that *MEKK1-MKK2-MPK4/MPK6* pathways positively regulate the cold response and chilling temperature tolerance in *Arabidopsis* (Teige et al., 2004). To induce the kinase mechanism, MKK2 activity is phosphorylated by MEKK1 under cold conditions (Raina et al., 2013), which further activates MKK2 that phosphorylates MPK4 and MPK6 (Teige et al., 2004), likely adjusting downstream molecules to regulate the cellular status. These findings showed that the *MMK2* pathway regulates the freezing response (Teige et al., 2004). *SlMPK3* upregulates the growth of tomato under low temperature (Yu et al., 2015). In potatoes, it is reported that the *MAPK3* showed a response under cold stress (Zhang et al., 2017) and chilling temperature stress (Xie et al., 2012). *MAPK3* also activates the phosphorylation downstream for ACC synthase and transcriptional factor OsbHLH002/OsICE1 for chilling outbreaks (Xie et al., 2012). Under cold stress, *MKK2-MPK4/MPK6* cascades are activated (Teige et al., 2004). In recent years, the MAPK genes in rice *OsMKK6-OsMPK3* have been identified, which are activated in cold stress (Fu et al., 2012). cDNA library screening revealed another well-studied MAPKK gene *OsMEK1* that showed interaction with *OsMAP1*, which is important in freezing temperature tolerance (An et al., 2012).

Under cold stress, the MPKK genes *ZmMKK4* (Wu et al., 2011) and *ZmMPK7*, as shown in **Figure 3** (Osthoff et al., 2019), showed upregulation in maize. These two genes are found in the nucleus and control the transcription factors at low temperature. Similarly, five more MAPKs have been identified, which responds positively in roots of maize under cold stress (Osthoff et al., 2019). It is described that the seedlings of cotton at low temperature (4°C) showed optimum growth against cold stress, especially in root cells because of the over-expression of the *MAPK* gene named *GhMAPK* (Zhang et al., 2011). *MAPK* genes in different crops like *Arabidopsis*, alfalfa, blue mustard (*Chorispora Bungeana)*, cotton, maize, rice, and sea bean (*Salicornia brachiate)* under cold and heat stress conditions are mentioned in **Table 3**. Cold stress causes severe damage in many regions of the world. Therefore, research should incorporate MAPK cascades with the help of modern technologies in breeding resistance cultivars of different crops by identifying novel *MAPK* gene families.

**Table 3:** MAPK gene signaling responses in different plants under cold and heat stress conditions.

Plant species	*MAPK* genes	Activation	References
Cold			
*Arabidopsis*	*AtMEKK1,6*	Calcium-dependent activation, to regulate vacuolar processing enzyme (VPE)	Zhang et al. (2006)
	*AtMPK1,6*	*MKK2* is the upstream activator of *MPK4* and *MPK6*	Zhang et al. (2018)
	*AtMAPKK, AtMKK2*		
Alfalfa	*MsSAMK, MsP44MKK4*	Activated by cold shock domain-containing proteins (CSDPs)	Agarwal et al. (2010)
Blue mustard	*CbMAPK3*	Activated under low temperature	Nakagami et al. (2005)
Cotton	*GhMAPK, GhMAPK2, 7*	Activated under 4°C	Yang et al. (2019)
Maize	*ZmMPK3,5*	Activated under high accumulation of proline	Zhang et al. (2011)
		
	*ZmMPK17*	Activated under chilling conditions	Kong et al. (2011)
Rice	*OsMSRMK2*	Activated at 12°C	Meng and Zhang (2013)
	*OsMAPKK2, 4,6* and *10*	Activated by low temperature stress at the transcriptional level	
	*OsMKK6, OsMPK3*	Activated by cold shock domain-containing proteins (CSDPs)	Xie et al. (2012)
	*OsMAPK5*	Activation under chilling temperature	Xiong and Yang, 2003
Sea bean	*SbMAPKK*	Activated by cold shock domain-containing proteins (CSDPs)	Meng and Zhang (2013)
Tomato	*SlMPK3*	Upregulation under low temperature	Yu et al. (2015)
Heat			
Rice	*OsMKK4,6*	Activated when temperature increased from 37°C	Wen et al. (2002); Kumar et al. (2008)
	*OsMSRMK2*	Activated at high temperature < 37°C	Agrawal et al. (2002)
Alfalfa	*MsHAMK*	Activated by the activation of heat shock protein.	Sangwan et al. (2002)
Potato	*StMPK1*	Activated by high temperature stress at the transcriptional level	Blanco et al. (2006)
Cucumber	*CsMKK4*	Activated by the upregulation of heat shock transcription factors (HSFs)	Wei et al. (2014)
Strawberry	*FvMAPKK3*	Activated by the upregulation of HSFs	Lohani et al. (2020)
Mulberry	*MnMAPK1,5,6*	Activated at 40°C under high temperature	Yan et al. (2016)
Tomato	*SlMPK1*	Negitively regulated under high temperature	Ding et al., 2018

### 4.3 The role of MAPKs under heat stress

Temperature is considered the most essential factor to achieve optimum metabolic processes, growth, development, and production of plants (Yin et al., 2014; Haider et al., 2022; Raza et al., 2022c). The amount of water in cells under different temperatures is a fundamental feature for cell survival, which is directly proportional to plant growth and damages the cell structure and organelles and reduces productivity (Jiang et al., 2015; Xing et al., 2015; Raza et al., 2021). High temperature also causes overproduction of ROS due to the high rate of respiration and photosynthesis, which significantly affects the growth and development of plants (Li et al., 2012). MAPKs play an important role under heat stress in plants such as aspen (*Brachypodium distachyon*); 60% of genes activated under high temperature by MAPKs signal transduction cascade (Suri and Dhindsa, 2008). To date, few studies about heat stress signaling of MAPKs provide evidence that only specific MAPKs are upregulated or activated under high temperature stress (Munns et al., 2020). It has been experimentally proven that *MAPK3* is activated under heat stress and ultra-irradiation and helps to mitigate stress by controlling the growth of plants (Tuteja, 2007).

Secondly, the heat shock protein HSP70 plays a vital role in response to heat stress signaling through the *HSP* gene by the activation of the MAPK named *HAMK* in tobacco (Wei et al., 2014). In cucumber (*C. sativus*), an *MAPK* gene named *CsMKK4* is activated after 8 h of heat treatment. Most of the *MAPK* genes showed activation under heat stress except *CsMPK3* and *CsMPK7* (Wei et al., 2014). The upregulated transcript level of *FvMAPK3*, *FvMAPKK1*, *FvMAPKK3*, *FvMAPKK6*, and *FvMAPKK7* in strawberry (*F. vesca*) has been observed under high temperature (Zhou et al., 2014). In the same way, under heat stress, the gene named *FvMAPKK3* is activated (Zhou et al., 2014). In mulberry (*morus*) under heat stress, eight MAPK genes have been identified, which showed upregulation (*MnMAPK1*, *MnMAPK5*, *MnMAPK6*, and *MnMAPK9*) and downregulation (*MnMAPK2*, *MnMAPK3*, *MnMAPK8*, and *MnMAPK10*) (Yan et al., 2016). Likewise, under high temperature, the downward growth regulation of the tomato gene named *SlMPK1* is shown (Ding et al., 2018). The above studies indicated that under heat stress conditions, MAPK cascade is regulated in plants. MAPK gene families related to heat resistance is negligible; due to climate change, this stress will be a severe threat in coming years. This stress can be overcome by identifying desirable *MAPK* gene families in commercial crops.

### 4.4 The role of MAPKs under salinity and osmotic stress

In plants, there are three major salt tolerance mechanisms, namely, osmotic pressure tolerance, ionic balance management, and decreasing the Na^+^ and Cl^−^ cytoplasmic concentrations (Mohamed et al., 2022; Raza et al., 2022d). Salinity stress is a very serious threat in many regions of the world; more than 50% of irrigated soils all over the world face reduction in the productivity of major agricultural crops (Shi et al., 2010). Under salt stress, cell membrane and protein disruption occurs due to the overproduction of ROS in plants, which is one of serious problems for crops grown under salt conditions (Nakagami et al., 2005; Raza et al., 2022d).

It was also reported that MAPK proteins are activated when plants are subjected to salt stress; this leads to speeding up the expression level of V-H^+^-ATPase, which increases the tolerance to salinity stress (Krasensky and Jonak, 2012). In *Arabidopsis*, a MAPKK gene named *MEKK1* mRNA accumulated in response to high salt stress. The protein–protein interaction between *MMK2/MEK1* and *MPK4* MAPKs, *MMK21* and *MKK2/MEK1* MAPKKs, and *MPK4* and *MEKK1* was shown by yeast two-hybrid analysis (Teige et al., 2004). It was also documented that salt stress signal transmission occurred at two MAPK cascades such as *MPK4* MAPK cascade with genes named *MEKK1-MEK1/MKK2-MPK4* and *MPK6* and a second MAPK cascade with a gene named *p44MAPK* involved in salt tolerance in *Arabidopsis*, as shown in **Table 4**. *MEKK1* involve as more upstream of *MKK2* and more downstream MAPKs *MPK4* and *MPK6* under salinity stress (Teige et al., 2004). MAKs such as *MPK6*, *MPK4*, and *MKP1* play a negative role under salinity stress conditions (Faried et al., 2017). A salt stress-induced MAPK named 46kDa *SIMK* showed response under salt stress in alfalfa (Krasensky and Jonak, 2012). *In vivo* as well as *in vitro* tolerance to salt stress produced by upstream kinase named *SIMKK* intermingles with *SIMK* by yeast two-hybrid (Krasensky and Jonak, 2012).

**Table 4:** MAPK gene signaling responses in different plants under salinity stress.

Plant species	*MAPK* genes	Activation	Concentration	References
*Arabidopsis*	*AtMEKK1,2*	Activation by accumulation of sodium chloride	High salt accumulation	Teige et al. (2004); Moustafa et al. (2008)
	*AtMPK1,3*	Activation of RD29A and RD29B gene promoters	Excess of salts	Yu et al. (2010)
	*AtMPK4,6*	Activated by phosphatidic acid (PA)	Acid accumulation	Moustafa et al. (2008)
Maize	*ZmMPK3,5*	Activated under very high salt concentration	High amount of salts	Wang et al. (2010)
	*ZmSIMK1*	Activated by high salinity	Higher salt accumulation	Gu et al. (2010)
	*ZmMKK4*	Activated by NaCl accumulation	Excess amount of NaCl	Kong et al. (2011)
Rice	*OsMSRMK2*, *MAPKK4, 6*	Activated in response to high salt and cytotoxicity.	NaCl accumulation	Agrawal et al. (2002)
	*OsMAPK5*,	Activated by salt accumulation	Very high concentration	Xiong and Yang (2003)
	*OsMPK3, 4, 5*	Activated by accumulation of arsenic stress and salts	High arsenic and salt concentration	Rao et al. (2010)
	*OsMKK6*	Activated in hypersensitivity of other stresses	High salinity	
	*OsEDR1*	Activated by high salt accumulation	Excess salt amount	Kim et al. (2003)
	*OsMAPK4*	Activated under salt accumulation	NaCl excess amount	Rao et al. (2010)
Cotton	*GhMAPK*, *GhMPK2*	Activated by salt stress and osmotic adjustment	High amount of salts	Wang et al. (2007); Zhang et al. (2011)
Blue mustard	*CbMAPK3*	Activated under salt accumulation	High salt accumulation	Zhang et al. (2006)
Sea bean	*SbMAPKK*	Activated under salt accumulation	Water reduction and salt accumulation	Agarwal et al. (2010)
Cucumber	*CsNMAPK*	Activated under salt accumulation	Salt accumulation	Xu et al. (2010)
Tobacco	*NtSIPK*	Activated by salt stress and osmotic adjustment	NaCl accumulation	Mikolajczyk et al. (2000)
Potato	*StMAPK3*	Activated by salt stress and osmotic adjustment	PEG, menthol, and NaCl treatment	Zhu et al. (2020)
Osmotic stress				
*Arabidopsis*	*AtMPK1, 4, 6*, and *20*	Activated under high salt accumulation and improved the osmosis	Hyper-osmolarity	Droillard et al. (2004); Moustafa et al. (2008)
	*AtMKK7,9*	Activated under higher NaCl concentration	Sodium chloride excess	Moustafa et al. (2008)
	*AtMPK9, 17,18*	Activated under higher salt accumulation	Hyper-osmolarity	Moustafa et al. (2014)
Maize	*ZmMPK7*	Activated under salt accumulation and molecular imbalance	Hyper-osmolarity	Zong et al. (2009)
Tobacco	*NtSIPK*	Activated by salicylic acid under high salt concentration	Higher osmotic stress	Mikolajczyk et al. (2000)
Alfalfa	*MsSIMK*	Upstream activation by *SIMKK*	Higher osmolarity	Jonak et al. (2004)
Potato	*StMAPK3*	Activated by NaCl (40 mM and 80 mM)	Salt accumulation	Zhu et al. (2020)

Crops respond to salt stress through different processes like elimination and appropriation of ions (Na^+^ and Cl^−^) into vacuole to lessen cytotoxicity; research has shown that osmotic stress is also regulated by MAPKs (Kim et al., 2003). Protective proteins like late embryogenesis abundant (LEA) and chaperones, a heat shocking protein also speed up to defense the negativity of these toxic ions. MAPKs also respond by conveying signals for osmotic stress to specific effectors and play a key role to survive in the cell under high salt concentration. It is already reported that MAPK cascade is activated under salt and osmotic stress at both transcriptional and protein levels (Iftikhar et al., 2017). In *A. thaliana*, genes like *AtMEKK1*, *AtMKK2*, and *AtMPK4*, as shown in **Figure 3**, have stress tolerance under salinity conditions. *MMK* gene named as *SIMKK* an upward regulator of *SIMK*, a *MAPK* gene response upregulation in alfalfa under salinity as well as osmotic stress (Xu et al., 2010). In addition, salicylic acid-induced protein kinase (*SIPK*) regulates osmotic stress in tobacco in a very short time (5 to 10 min) (Ghorbel et al., 2019; Habiba et al., 2021); *SIPK* is an *Arabidopsis MPK6* homolog in tobacco.

In another study, it was also shown that plant growth recovered in the overexpression lines of *StMAPK3* in potato, and the lethal effects of osmotic stress and salinity were reduced by the MAPK gene family (Zhu et al., 2020). The increased content of many oxidative markers like NaCl, H_2_O_2,_ polyethylene glycol (PEG), and menthol is weakened by *StMAPK3* overexpression in potato (Zhu et al., 2020). Similarly, the opposite effect is shown by catalase (CAT), peroxidase (POD), superoxide dismutase (SOD), and proline content by *StMAPK3.* In *Arabidopsis*, interchangeable names *MPK4* and *MPK6* have been identified, whereas salinity signal responding genes like *MEKK1* and *MKK2* have also been recognized (Teige et al., 2004). Salt tolerance is shown by the stress marker gene *MKK2* in transgenic plants. Gene promoters called RD29A and RD29B were activated by the expression of *MKK* and *MPK*, respectively (Zaheer and Akhtar, 2016), which shows that the MAPKs regulate upward under salinity and osmotic stress. Many MPKs (*MPK9*, *MPK10*, *MPK11*, *MPK17*, and *MPK18*), MKKs (*MKK7* and *MKK9*), and MEKKs (*MEKK3*, *MEKK5*, *MEKK6*, and *MEKK7*) have been screened for salt and osmotic stress (Moustafa et al., 2008; Chen et al., 2015). In maize, the MAPK named *ZmSIMK1* was identified as having high salt tolerance by the overexpression of *Arabidopsis* transgenic plants. In the same way, in maize, *ZmMPK17* also showed high osmotic stress tolerance by the activation of *Arabidopsis MPK17* (Yang et al., 2019), which provides an idea that further description and practical study of *MPK17* and its ortholog can provide better achievement in abiotic stress tolerance gene identification and screening.

It is also described that, in rice, MAPKs control salinity and osmotic stress; *OsMPK4*, *OsMPK3*, *OsMSRMK2*, *OsEDR1*, *OsEDR1*, *OsMAPK5*, and *OsMAPK4* have been classsified as salinity tolerance genes (Xie et al., 2012). Salt tolerance and osmotic pressure are also affected by the activation of *OsMPK5* and *OsMPK4* in transgenic maize line (Xiong and Yang, 2003). Over-expressing *GhMPK2* shows the osmotic stress tolerance in transgenic cotton (*G. hirsutum*) (Yang et al., 2019). Similarly, cucumber roots and transgenic tobacco seeds showed overexpression of MAPK genes called *CsNMAPK* and *CsNMPAK*, which showed a germination rate higher than wild type (Gomi et al., 2005), which proves that *CsNMPAK* performs better under salt stress condition in the early stages. Salinity is associated with osmotic stress; from the above studies, it is strongly depicted that MAPK genes that are activated under salinity conditions are also upregulated under osmotic stress conditions. The MAPK genes in different crops like *Arabidposis*, maize, rice, cotton, blue mustard, alfalfa, rice, tobacco, potato, sea bean, cucumber, tobacco, and potato under salinity stress and *Arabidposis*, maize, tobacco, alfalfa, and potato under osmotic stress are mentioned in **Table 4**.

The above discussion revealed that many MAPK genes related to salinity and osmotic stress had been identified, and highly expressed under saline and osmotic stress conditions. By identifying more MAPK genes in crops, crop yield and productivity can be improved, and saline soil can be brought under cultivation.

### 4.5 The role of MAPKs under heavy metal stress

Heavy metal ions play a vital role in the development and growth as well as in the physiological processes of plants (Lee and Ellis, 2007; Raza et al., 2022e). However, at higher concentrations, they can have lethal effects on plant physiology. With the increase in concentration of heavy metals in the soil, the cellular response of the plants is activated (Raza et al., 2022e). A novel type of *MAPK* gene called *OsMSRMK2* in rice is activated when plants have high levels of cadmium, mercury, and copper ions (Yang et al., 2019). Studies have also confirmed the activation of *MAPK* genes in rice (*O. sativa*) with the high level of cadmium ions (Haider et al., 2021).

Many *MAPK* genes like *SIMK*, *MMK2*, *MMK3*, and *SAMK* in alfalfa (*M. sativa*) seedling are activated when they are exposed to copper and cadmium ions stress (Gupta et al., 2009). Studies have also confirmed that the activation of SIMKK is only under copper ion stress but not under cadmium ion stress, as shown in **Table 5**. It was documented that this MAPK activity is specific in response as specific treatment indicates the specific signals’ transduction (Yeh et al., 2004). Involvement of *MAPK* genes called *OsMPK3*, *OsMPK4*, and *OsMKK4* mediated heavy metal stress tolerance in rice seedlings as shown in **Figure 3** (Gupta et al., 2009). In maize (*Z. mays*), under heavy metal stress, the activation of *MAPK* genes has also been confirmed (Lumbreras et al., 2010). These findings show the role of MAPKs in signaling activation under various heavy metal stresses.

**Table 5:** MAPK gene signaling responses in different plants under heavy metal stress conditions.

Plant species	*MAPK* genes	Stress causing metals	Methods	Reference
Alfalfa	*MsMMK2, MsMMK3, MsSAMK*	Cadmium and copper	Complex activation pattern of *MAPKs: SIMK, MMK2, MMK3*, and *SAMK*	Jonak et al. (2004)
Rice	*OsMSRMK2, OsMPK3,4*, and *OsMKK4*	Mercury, copper, cadmium, and arsenic	Overexpression of functions of an *OsMSRMK2* and *MBP kinase*	Agrawal et al. (2002)
	*OsHMA3*	Cadmium	Overexpression of a functional allele of *OsHMA3*	Ueno et al. (2010); Shao et al. (2018)
	*OsLCT1*	Cadmium	Knockdown of *OsLCT1*	Ueno et al. (2010)
	*OsNRAMP5*	Cadmium	CRISPR/Cas9-mediated editing of *OsNRAMP5*	Uraguchi et al. (2011)
	OsHMA3	Cadmium	Articulation and tissue restrictions of OsHMA3	Shao et al. (2018)
Maize	*ZmMPK3*	Cadmium	Signaling activation of *ZmMPK3*	Wang et al. (2010)

From the above discussion, except rice, maize, and alfalfa, there are no other crops in which *MAPK* gene families have been identified. In the near future, heavy metal stress will severely threaten crops due to climate change and heavy industrialization. The researcher’s job is to verify the functional roles of *MAPK* genes and other mechanisms to develop heavy metal resistance cultivars.

## 5 Conclusion

There is growing evidence that the MAPK cascade is the hub of a sophisticated network structure that transduces signals related to plant stress tolerance. The MAPK cascade gradually amplifies and conveys stress signals to downstream response components through phosphorylation and dephosphorylation, leading to various stress responses. Furthermore, a better understanding of the MAPK cascades’ process should make it easier to create new methods for enhancing plants’ ability to withstand stress. Numerous genetic engineering methods are available to increase abiotic stress tolerance in crops. As key players in signal transduction and regulators of gene transcription, MAPK cascades have already been used to improve abiotic stress tolerance, as discussed in this review. Molecular biology analysis of the MAPK cascade’s components and its function is essential for enhancing crop improvement. Research on the function of MAPK genes or the mechanism by which the MAPK cascade regulates plant stress resistance is still limited, despite many studies in plants that have demonstrated the involvement of MAPK cascades in numerous biological processes in response to abiotic and biotic stresses. In addition, different stress stimuli can activate the same MAPK cascade genes. The above discussion concludes that the MAPK cascades and the molecular mechanisms of plant stress resistance have great significance for elucidating the entire stress tolerance signal transduction pathway in plants.

## 6 Future perspective

Abiotic stress in crops helps to expand the tolerance of crops under different abiotic stresses by various methods of genetic manipulation. A number of studies recounted that by exploiting MAPK pathways in crops like *Arabidopsis* and many other crops like potato, maize, rice, and poplar, stress tolerance has improved. In the near future, investigation should include detecting the MAPK substrate, and by using advanced breeding methods and molecular methodologies, the development of new lines would be able to withstand harsh environments to meet the food requirements of the increasing population. Interacting protein is the main factor that needs to identify and quantify for producing multiple environmental stress, also with their mode of action, which is like that (MAPKKK-MAPKK-MAPK) chain and accumulated into one functional ‘MAPK transgenic circuits’ which could be inserted into target sequence or cell through genetic engineering and biotechnology to develop a tolerance species for specific function having specific kinase protein.

It is important to study the function of MAPKs related to abiotic stress in crops, and previous studies have confirmed their role in crops related to abiotic as well as biotic stress. In the future, there is a need to identify more *MAPK* gene families in crops related to environmental stresses and also identify their functional analysis through advanced methodologies like transcriptomics, proteomics, metabolomics, bioinformatics, CRISPR/Cas technology, and DNA/RNA sequencing to encourage analysis of a regulation network that controls abiotic stress response. In addition, identification and functional analysis of MAPKs can be further processed by mutation, gene silencing, and microRNA techniques to produce MAPK mutants and genetically engineered gene families, which transform into crops for tolerance to biotic stresses.

## Author contributions

YM, HS, and XZ conceived the idea. YM, XZ, NZ, Nu-A, AR, and FH contributed to writing and literature search. YM and Nu-A contributed to organizing tables and figures. All authors contributed to the article and approved the submitted version.
